# Electronic Cigarette Exposure Enhances Lung Inflammatory and Fibrotic Responses in COPD Mice

**DOI:** 10.3389/fphar.2021.726586

**Published:** 2021-07-28

**Authors:** Hongwei Han, Guangda Peng, Maureen Meister, Hongwei Yao, Jenny J. Yang, Ming-Hui Zou, Zhi-Ren Liu, Xiangming Ji

**Affiliations:** ^1^ Department of Biology, Georgia State University, Atlanta, GA, United States; ^2^ Division of Pulmonary and Critical Care, Department of Medicine, Massachusetts General Hospital, Harvard Medical School, Boston, MA, United States; ^3^ Department of Nutrition, Georgia State University, Atlanta, GA, United States; ^4^ Division of Biology and Medicine, Department of Molecular Biology, Cell Biology and Biochemistry, Brown University, Providence, RI, United States; ^5^ Department of Chemistry, Georgia State University, Atlanta, GA, United States; ^6^ Center for Molecular and Translational Medicine, Georgia State University, Atlanta, GA, United States

**Keywords:** ENDS, COPD, M2 macrophage, lung fibrosis, inflammation

## Abstract

Although a few studies show that the use of electronic nicotine delivery systems (ENDS) may ameliorate objective and subjective outcomes in COPD smokers who switched to electronic cigarettes, it is unclear whether e-cigarette exposure alters lung pathological features and inflammatory response in COPD. Here, we employed βENaC-overexpressing mice bearing COPD-like pulmonary abnormality, and exposed them to ENDS. We found that ENDS exposure aggravated airspace enlargement and mucus production in βENaC-overexpressing mice, which was associated with increased MMP12 and Muc5ac, respectively. ENDS exposure to mice significantly increased the numbers of macrophages, particularly in M2 macrophages in bronchoalveolar lavage (BAL) fluid, despite ENDS did not induce M2 macrophage polarization in a cultured murine macrophage cell line (RAW264.7). There were no changes in neutrophils in BAL fluid by ENDS exposure. Multiple cytokine productions were increased including M-CSF, IL-1rα, IL-10, and TGF-β1, in BAL fluid from mice when exposed to ENDS. The Sirius Red staining and hydroxyproline assay showed ENDS-exposed mice displayed enhanced fibrotic phenotypes compared to control mice. In conclusion, ENDS exposure enhances airspace enlargement, mucus secretion, and fibrogenesis in COPD mice. This is associated with increased MMP12, inflammatory responses, and M2 macrophage phenotype. This study provides pre-clinical data implicating that electronic cigarette exposure is not safe in COPD patients who want to replace traditional cigarettes with ENDS.

## Introduction

Electronic nicotine delivery systems (ENDS), also referred to as e-cigarettes, are electronic devices that produce an aerosol by heating a liquid that contains nicotine predominantly (Breland et al., 2017). ENDS is generated for stopping traditional cigarette smoking that is a major risk factor for chronic obstructive pulmonary disease (COPD). Some studies show that ENDS use may ameliorate objective and subjective outcomes in COPD smokers who switched to e-cigarettes, which is associated with abstinence and conventional smoking reduction (Polosa et al., 2016; Polosa et al., 2020). However, ENDS is not safe. Emerging evidence shows that ENDS trigger severe lung injuries and lead to the development of various lung diseases (Shields et al., 2017; Gilpin, 2019; Bhatt et al., 2020; Bhatta and Glantz, 2020; Mcalinden et al., 2020; Traboulsi et al., 2020). In 2019, an epidemic of E-cigarette-associated lung injury affected 49 states of the United States with over 2,600 cases and 60 deaths reported (Belok et al., 2020; Cherian et al., 2020). Moreover, growing evidence reveals that ENDS use is directly associated with the diagnosis of COPD in humans in recent years, and the exposure to ENDS is reported to induce COPD-like phenotype in mice (Garcia-Arcos et al., 2016; Bowler et al., 2017; Perez et al., 2019; Xie et al., 2020). However, the mechanisms have not been fully uncovered yet.

COPD is a primary leading cause of death affecting an estimated 328 million worldwide (Eisner et al., 2010; Quaderi and Hurst, 2018). It is characterized by progressive and irreversible airflow obstruction caused by chronic lung inflammation, in which innate immune cells play a pivotal role (Rovina et al., 2013; Barnes, 2016). Especially, macrophages are key effector cells in COPD which orchestrate immune responses (Kapellos et al., 2018). Macrophages are observed remarkedly increased in lung tissues and bronchoalveolar lavage **(**BAL) fluid of COPD patients and are associated with COPD severity (Hiemstra, 2013). In general, macrophages consist of two major phenotypes (Mosser and Edwards, 2008). M1 macrophages, referred to as classically activated macrophages, have been described as cytotoxic and pro-inflammatory, and are characterized by secretion of pro-inflammatory cytokines such as interferon (IFN)-γ, IL-6, and IL-12 (Mantovani et al., 2004; Mantovani et al., 2013). M2 macrophages, referred to as alternatively activated macrophages, are considered anti-inflammatory and are related to tissue repair and fibrosis, producing majorly anti-inflammatory cytokines including transforming growth factor β1 (TGF-β1), IL-4, IL-10, M-CSF (Gordon and Martinez, 2010; Martinez and Gordon, 2014; Chávez-Galán et al., 2015). Two phenotypes can switch between each other by different stimuli (Das et al., 2015; Funes et al., 2018). Multiply research groups have reported an alteration of immunophenotypes of macrophages towards M2 phenotype in COPD subjects, with cytokine production skew towards an M2 profile in blood and BAL samples from patients, suggesting M2 macrophages phenotype is a critical player in COPD disease progression (Eapen et al., 2017a; Eapen et al., 2017b; Da Silva et al., 2020). However, it is unclear whether macrophages undergo phenotype alterations after ENDS exposure in COPD, understanding of which would be of great importance to uncover the impact of ENDS on COPD.

It has been shown that βENaC-overexpressing mice have COPD-like pulmonary abnormalities, including mucous hypersecretion, inflammatory and emphysematous phenotypes and pulmonary dysfunction (Johannesson et al., 2012; Seys et al., 2015; Shuto et al., 2016). In this study, we employed βENaC-overexpressing mice and exposed them to ENDS to determine whether ENDS aggravate or exacerbate COPD-like pathological changes. We also measured macrophage immunophenotype and lung fibrogenesis in these mice exposed to ENDS. We observed a significant exacerbation of COPD features in these mice, along with a remarkable change in multiple cytokine production in COPD mice after exposure to ENDS. Further investigations elucidated that the ENDS challenge increased the total macrophage population but did not change neutrophil numbers. Macrophage phenotype was altered with an increase in M2 phenotype after ENDS exposure. In addition, fibrotic phenotypes were enhanced in ENDS-exposed mice, evidenced by a dramatic increase in the level of pro-fibrotic cytokine TGF-β1 and an augment in collagen production. For the first time, our study shows that the ENDS affect macrophage phenotype alteration during COPD development in mice, which leads to an enhancement of lung fibrosis.

## Materials and Methods

### Animal Model

βENaC mice with C57BL/6J background were obtained from the Jackson Laboratory. All animal procedures described were approved by the Institutional Animal Care and Use Committee (IACUC) at Georgia State University. βENaC mice were bred with wild-type C57BL/6J mice to produce hemizygous βENaC for our studies. Animals were genotyped to determine the genetic overexpression of *Scnn1b*. Briefly, tail snips were harvested and lysed in Proteinase K at 56°C water bath overnight. DNA was extracted by Dneasy Blood and Tissue kit (Qiagen, Foster City, CA). The overexpression of the mouse *Scnn1b* gene was identified by PCR (Forward: CCT​CCA​AGA​GTT​CAA​CTA​CCG; Reverse: TCT​ACC​AGC​TCA​GCC​ACA​GTG) (Mall et al., 2004). After PCR, samples were run on a 4% agarose gel containing ethidium bromide. Mice carrying *Scnn1b* overexpression were tagged as βENaC mice for future studies.

### Electronic Nicotine Delivery Systems Exposure to βENaC Mice

**FIGURE 1:**
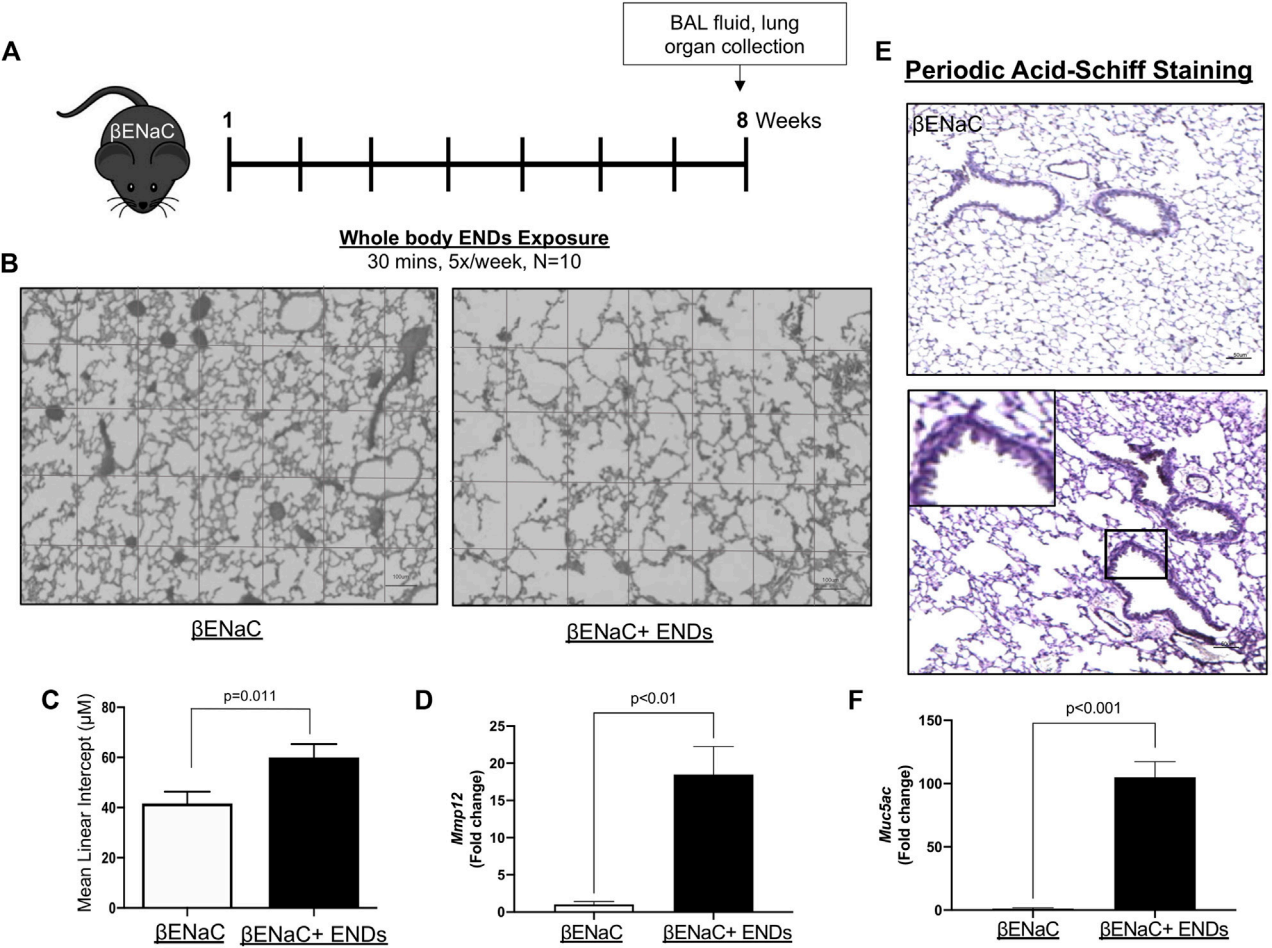
E-cigarette exposure aggravates COPD in βENaC mice. **(A)** Experimental schematic of whole body ENDS exposure in βENaC mice. **(B)** ENDS exposure caused emphysematous phenotypes changes in βENaC mice. Representative data of H&E staining images using lung sections of sham air (left) (*n* = 4) or ENDS exposed (right) (*n* = 4) mice. **(C)** Quantification of the free distance between gas exchange surfaces in lungs of βENaC mice with or without ENDS exposure. **(D,F)** Determination of mRNA levels of COPD-related proteins MMP12 **(D)** and Muc5ac **(F)** in lung tissues from βENaC mice with or without ENDS exposure by qPCR. **(E)** Representative images of Periodic Acid-Schiff staining in lung sections of βENaC mice with or without ENDS exposure. The inset indicates the enlarged image of the boxed area showing positive staining. All data presented at mean ± SEM.

At 12-week of age, mice (*n* = 10/group) were randomized into either the ENDS exposure group or the control group. Mice in the ENDS group were exposed to e-cigarette vapor for 30 min daily at a frequency of 5 times per week for a total duration of 8 weeks as shown in Figure 1A. Mice were placed in a smoking chamber attached to an inExpose Smoking Robot (SCIREQ, Montreal, QC, Canada) with an attached e-cigarette accessory (ECX JoyeTech E-Vic Mini, SCIREQ, Canada) for the administration of vaporized S brand e-cigarette liquid containing nicotine at 50 mg/ml with caramel flavoring. After 8 weeks of e-cigarette vapor exposure, animals were euthanized within 24 h after the last e-cigarette vapor exposure using CO_2_. Then, bronchoalveolar lavage (BAL) fluid, and lung tissues were collected for analysis as described below.

### Cell Counts and Cytokine Measurements in Bronchoalveolar Lavage Fluid

After the mice were euthanized, tracheostomy was performed, and lungs were immediately flushed with 1 ml ice-cold phosphate-buffered saline (PBS) twice for collection of BAL fluid. Total cells from the BAL were centrifuged and counted using Countess™ II Automated Cell Counter (Invitrogen, Carlsbad, CA) as previously described (Mall et al., 2004). Total immune cell count (5,000 cells/slide) was performed on Shandon cytospin slides (Thermo Shandon, Pittsburgh, PA) stained with Diff-Quik (Dade Bering, Newark, DE). The BAL supernatants were stored at −80°C until analysis.

### Morphometric Assessment

In order to determine the effects of ENDS exposure on the development of emphysema, we did a morphometric assessment according to the previous protocol (Bartalesi et al., 2005; Mall et al., 2008). Briefly, the mouse lungs were inflated with 1% low melting-point agarose to 25 cm of H_2_O pressure. After 48 h of fixation, lungs were paraffin-embedded, sectioned, and stained with hematoxylin and eosin (H&E). The paraffin slides were baked at 60°C for 2 h, then deparaffinized with xylene and rehydrated with graded ethanol solutions. Next, the slides were incubated with Mayer’s Hematoxylin for 10 min and washed under tap water for 10 min. Slides were then immersed in Eosin for 30 s and washed under tap water, after which, the slides were dehydrated and mounted with a mounting medium. Mean linear intercepts were determined and calculated (Dunnill, 1962).

### Immunohistochemistry

The mouse lungs were inflated with 1% low melting-point agarose to 25 cm of fixative pressure and then fixed with 4% neutral buffered formalin for 48 h. Later, these tissue samples were embedded in paraffin, and sectioned into 4 µm sections using a rotary microtome. For immunohistochemical analysis, tissue sections were deparaffinized with xylene and rehydrated with graded concentrations of ethanol, followed by antigen retrieval in 10 mM citric acid solution (pH = 6) for 20 min using a pressure cooker. Endogenous peroxidase activity was blocked by 3% hydrogen peroxide. After blocking with 5% BSA for 30 min, the slides were incubated with the primary antibodies against the following proteins at 4°C overnight: α-smooth muscle actin (α-SMA) 1:500 (Ab5694; Abcam); Arginase I 1:1,000 (sc-20150; Santa Cruz). After washing, the sections were incubated with the appropriate HRP polymers and developed with 3–3′ diaminobenzidine solution (DAB substrate kit; Vector Laboratories). After counterstaining with hematoxylin, the slides were dehydrated and mounted with a mounting medium.

### Periodic-Acid Schiff Staining

Periodic-acid Schiff (PAS; Sigma Aldrich, Saint Louis, MO) staining was used to evaluate mucus accumulation and immune cell infiltration. The Periodic-acid Schiff staining was performed following the manufacturer’s instructions. All images were taken at 20x magnification using Keyence Fluorescent Microscope (Keyence, Itasca, IL).

### Sirius Red Staining

Sirius Red staining was performed with Novaultra Sirius Red Stain Kit (IW-3012, IHC world) following the instructions of the manufacturer. In brief, the paraffin-embedded slides were deparaffinized in xylene and hydrated in gradient concentrations of ethanol. The slides were stained with Weigert’s hematoxylin for 8 min, followed by picro-sirius red for 1 h. After two washes in acidified water, slides were dehydrated, mounted in a resinous medium, and covered by cover slides.

### Cell Culture

Raw264.7 cells were purchased from ATCC and cultured in DMEM media supplied with 10% fetal bovine serum. Raw264.7 cells were maintained in a cell culture incubator at 37°C and 5% CO_2_. To test whether ENDS can induce macrophage polarization, Raw264.7 were incubated with DMSO or ENDS with nicotine (50 mg/ml) or IL-4+IL-13 (IL-4 20 ng/ml; IL-13 20 ng/ml) for 48 h and harvested for analysis of protein expression by western blot.

### Flow Cytometry

Freshly harvested lung tissues were minced and dissociated using collagenase D (Sigma 11088858001, 0.5 mg/ml) and DNase I for 20 min at 37°C on a shaker. Cell suspensions were subsequently passed through a 40 μm cell strainer to get a single cell suspension. Non-specific Fc-mediated interactions were blocked by incubating cells with CD16/CD32 antibodies (Thermofisher, 14–0161–82). Cells were labeled with fluorophore-conjugated antibodies, including CD45-FITC (BioLegend 103,108), F4/80-PE/Cy5 (BioLegend, 123111), CD11b-APC (Thermofisher, 17–0112–82), Ly6G-BV421(BioLegend, 127627), CD206-e450 (Thermofisher, 48–2061–82). At the last step, Cells were resuspended in FACS staining buffer and acquired using LSRFortessa flow cytometer (BD Biosciences). Data were analyzed by FlowJo software (Tree Star).

### RNA Isolation and RT-qPCR Analysis

The expressions of inflammatory cytokines, proteins, and growth factors were assessed with qPCR using lung tissues. The total RNA was isolated using the RNeasy Plus mini kit (Qiagen) according to the manufacturer’s instructions. For RT-qPCR, 0.5 μg of total RNA was reverse-transcribed to cDNA using Maxima First Strand cDNA Synthesis Kit (Thermo Fisher K1641). Real-time qPCR was then performed using 2 μL of cDNA using Luna Universal qPCR Master Mix (NEB M3003) with SYBR^®^ Green as the fluorescent dye and the 7,500 Fast Real-Time PCR System (Life Technologies). The primer pairs are used at a final concentration of 250 nM. Primer sequences are listed in the box below (Table 1). Cyclophilin was used as the reference gene. The relative transcript abundance of target genes compared with the reference gene was expressed in the cycle threshold (ΔCt) as ΔCt = Ct(target)—Ct(reference). The relative difference in transcript levels of the treated group compared with the control group was expressed as ΔΔCt = ΔCt(treated)—ΔCt(control). The relative fold changes in the transcript level were represented as 2−^ΔΔCt^.

**TABLE 1:** PCR primers used in this study.

Gene	Species	Forward sequence	Reverse sequence
Cyclophilin	Mouse	CTT​CGA​GCT​GTT​TGC​AGA​CAA AGT	AGA​TGC​CAG​GAC​CTG​TAT​GCT
SCNN1B	Mouse	CCT​CCA​AGA​GTT​CAA​CTA​CCG	TCT​ACC​AGC​TCA​GCC​ACA​GTG
M-CSF	Mouse	ATG​AGC​AGG​AGT​ATT​GCC​AAG​G	TCC​ATT​CCC​AAT​CAT​GTG​GCT​A
IL-1Rα	Mouse	GCT​CAT​TGC​TGG​GTA​CTT​ACA​A	CCA​GAC​TTG​GCA​CAA​GAC​AGG
IL-10	Mouse	GCT​CTT​ACT​GAC​TGG​CAT​GAG	CGC​AGC​TCT​AGG​AGC​ATG​TG
MMP12	Mouse	CGC​AGC​TCT​AGG​AGC​ATG​TG	TGG​GCT​AGT​GTA​CCA​CCT​TTG
Muc5ac	Mouse	GGA​CTT​CAA​TAT​CCA​GCT​ACG​C	CAG​CTC​AAC​AAC​TAG​GCC​ATC
TGFβ-1	Mouse	CTC​CCG​TGG​CTT​CTA​GTG​C	GCC​TTA​GTT​TGG​ACA​GGA​TCT​G

### Data Analysis

In the case of only two groups, two-tail *t*-tests were performed to assess the differences. All analyses were carried out using Prism7.0 software (GraphPad Software Inc., San Diego, CA). Values are represented as mean ± SEM. Levels of significance designated as *p* < 0.05 unless otherwise indicated.

## Results

### E-Cigarette Exposure Aggravates Chronic Obstructive Pulmonary Disease in βENaC Mice

In our study, the βENaC mice were given whole-body ENDS exposure as indicated (Figure 1A). At the end of the treatment, the mice were sacrificed, and organs were collected for further analysis. We observed that lung morphology was changed, and the lung normal structure integrity was damaged after ENDS exposure (Figure 1B). Further assessment of airspace size by mean linear intercept indicated that the free distance between gas exchange surfaces was significantly increased in ENDS-exposed mice by more than 40%, indicating that there were emphysematous changes to lungs (Figures 1B,C). Moreover, we observed a significant accumulation of mucus in bronchi after ENDS exposure (Figure 1E). Additionally, the expression levels of Muc5ac and Mmp12, which are highly involved in COPD (Caramori et al., 2009; Hunninghake et al., 2009; Churg et al., 2012; Li and Ye, 2020), were also increased after ENDS exposure (Figures 1D,F). Overall, the lung emphysema and mucus overproduction after ENDS exposure suggested ENDS aggravate COPD phenotype in βENaC mice background.

### E-Cigarette Exposure Elevates the Inflammatory Response

**FIGURE 2:**
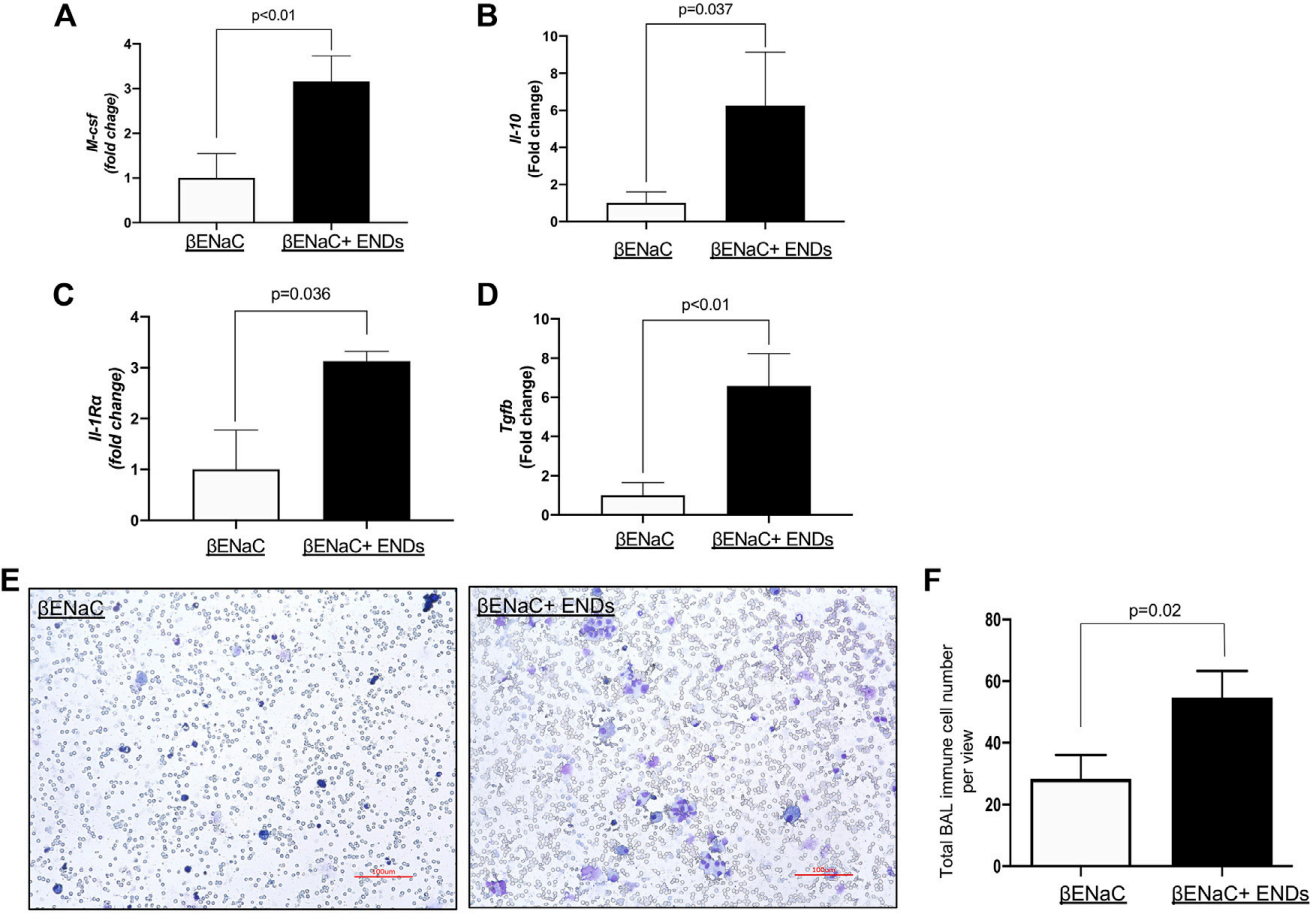
E-cigarette exposure elevates inflammatory response. **(A–D)** Determination of mRNA levels of inflammatory cytokines in lung tissues from βENaC mice with or without ENDS exposure by qPCR. **(E)** Representative images of Diff-Quik staining of BAL from βENaC mice with or without ENDS exposure (*n* = 4 per group). **(F)** Quantification of Diff-Quik staining as in (E) (*n* = 4 per group). All data presented at mean ± SEM.

COPD is characterized by lung inflammation, leading to progressive and irreversible airflow obstruction (King, 2015). Next, we determined the cytokine production in mice with or without exposure to ENDS. We measured the production of major cytokines by real-time qPCRs using lungs from mice of different groups. The results showed that the ENDS exposure significantly increased the production of multiple cytokines, including M-CSF, IL-1rα, IL-10, and TGF-β1(Figures 2A–D). In addition, the number of immune cells in BAL fluid collected from mice with ENDS exposure was remarkedly higher than that from mice without ENDS exposure (Figures 2E,F). Together, the ENDS elevate multiple cytokine production, indicating a skewed inflammation response.

### E-Cigarette Increases Macrophage Numbers in Lungs

**FIGURE 3:**
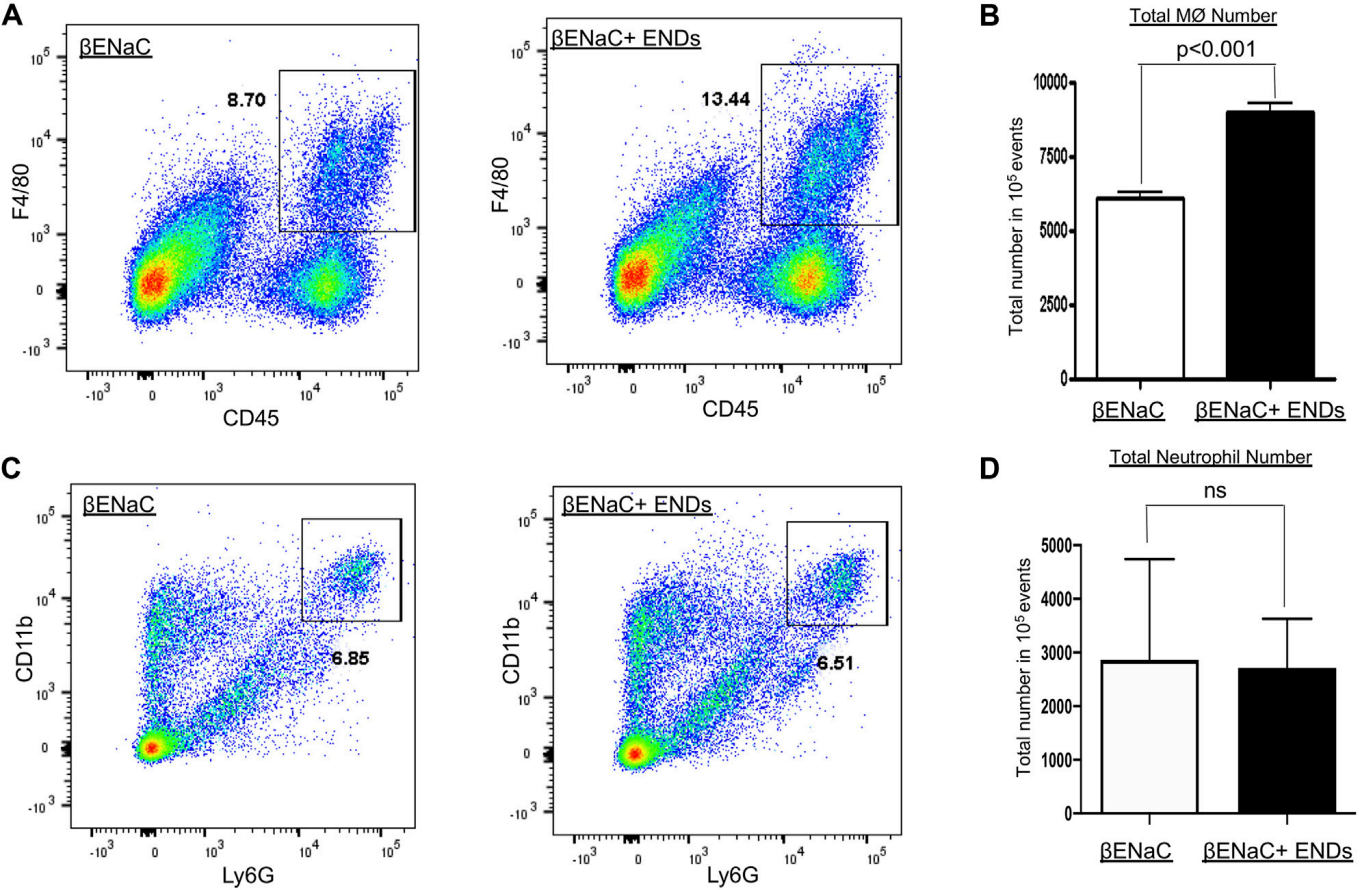
E-cigarette increases macrophage numbers in lungs. **(A,C)** FACS analysis of cell population of CD45^+^F4/80^+^ macrophages and CD11b^+^Ly6G^+^ neutrophils in lung tissues of βENaC mice with or without ENDS exposure (*n* = 3 per group). **(B,D)** Quantification of FCAS analysis of macrophage and neutrophil population as in **(A)** and **(C)**, respectively. All data presented at mean ± SEM.

Since ENDS exposure significantly augmented levels of cytokines, which are predominantly secreted by immune cells, for instance, macrophages, so our next question was asked as to whether ENDS exposure modulated populations of immune cells. We then assessed the numbers of macrophages and neutrophils with FACS and found that ENDS exposure increased the numbers of macrophages (Figures 3A,B), but did not change that of neutrophils (Figures 3C,D).

### E-Cigarette Increases the Population of M2 Phenotype of Macrophages

**FIGURE 4:**
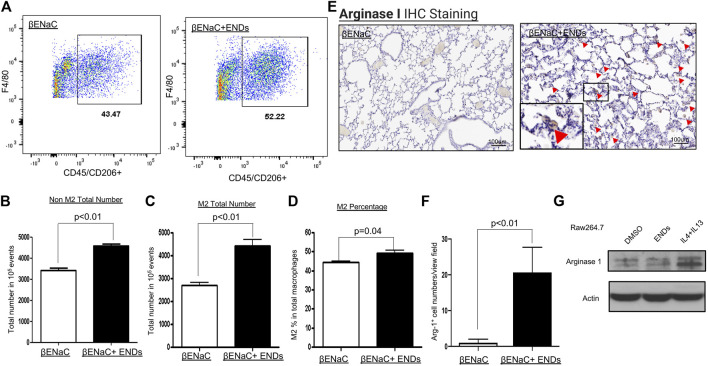
E-cigarette increases the population of M2 phenotype of macrophages. **(A)** FACS analysis of the cell population of F4/80^+^CD206^+^ M2 macrophages in lung tissues of βENaC mice with or without ENDS exposure (*n* = 3 per group). The M2 macrophages were pre-gated on live CD45^+^ cells. **(B)** Quantification of FACS analysis of CD45^+^F4/80^+^CD206^-^ non-M2 macrophage cell number in lung tissues of βENaC mice with or without ENDS exposure. **(C)** Quantification of FACS analysis of CD45^+^F4/80^+^CD206^+^ M2 macrophage cell number in lung tissues of βENaC mice with or without ENDS exposure. **(D)** Quantification of FACS analysis of CD206^+^CD45^+^F4/80^+^ M2 phenotype percentage in CD45^+^F4/80^+^ total macrophages in lung tissues of βENaC mice with or without ENDS exposure. **(E)** Representative images of immunohistochemical (IHC) staining of Arginase I in lung tissues of βENaC mice with or without ENDS exposure. The inset indicates the enlarged image of the boxed area showing positive staining. **(F)** Quantification of IHC staining of Arginase I as in **(E)**. The positive staining areas were calculated as percentages of total area, using five randomly selected sections per mouse, a total of four mice in each group. **(G)** Representative blots showing the expression level of Arginase I after indicated treatments. Actin was used as an internal control. Raw264.7 were treated with DMSO or ENDS (50 mg/ml) or IL-4+IL-13 (IL-4 20 ng/ml; IL-13 20 ng/ml) for 48 h and harvested for analysis of protein expression by western blot. Columns and error bars represent means ± SEM.

Macrophages have two subtypes, M1 and M2. M1 and M2 macrophages have different functions and distinct cytokine profiles. The qPCR results showed ENDS promoted the productions of multiple cytokines including M-CSF, IL-1rα, IL-10, and TGF-β1. Notably, IL-1rα, IL-10, and TGF-β1 are typical cytokines of the M2 phenotype of macrophages, especially IL-10, which is predominantly secreted by M2 macrophages (Martinez and Gordon, 2014; Da Silva et al., 2015; Rőszer, 2015). Therefore, we wondered whether ENDS exposure affected the M2 phenotype population. We used FACS to separate the non-M2 and M2 macrophage populations. The single cells were dissociated from lung tissues treated with and without ENDS and labeled by CD45, F4/80, and CD206. M2 macrophages were identified as F4/80^+^CD45^+^CD206^+^ population while non-M2 macrophages as F4/80^+^ CD45^+^CD206^-^ population (Figure 4A). The results showed that both non-M2 and M2 populations were significantly increased by ∼25 and ∼50% respectively after ENDS exposure (Figures 4B,C). Notably, the increase in the M2 population was larger compared to the increase in the non-M2 population. We further quantified the percentage of M2 in total macrophages and found that the percentage of M2 in total macrophages was remarkably elevated after ENDS treatment (Figure 4D). In addition, we further assessed M2 macrophages by performing immunohistochemical staining of Arginase 1, a commonly used marker for M2 macrophages, in lungs from mice with and without ENDS exposure. The results showed that the staining of Arginase 1^+^ M2 macrophages was significantly enriched in ENDS-treated mice (Figures 4E,F). Therefore, our results suggested ENDS exposure resulted in a phenotype alteration of macrophages towards M2 in mouse lungs.

Since we observed that ENDS increased the M2 macrophage population, then we asked whether ENDS directly induced M2 macrophages polarization. We tested our hypothesis using murine macrophage cell line Raw264.7. We treated Raw264.7 with DMSO, ENDS, or IL-4+IL-13 and then harvested cells for immunoblots of M2 marker Arginase 1. IL-4+IL-13, which are well-known to induce M2 macrophage polarization, are used as a positive control. Immunoblot results demonstrated that ENDS treatment did not increase Arginase 1 expression compared to DMSO treatment, while IL-4+IL-13 upregulated expression of Arginase 1 in Raw264.7 (Figure 4G). Our results suggest that ENDS do not induce M2 macrophage polarization directly in the cultured murine macrophage cell line.

### E-Cigarette Exposure Enhances Fibrotic Phenotypes in the Lungs

**FIGURE 5:**
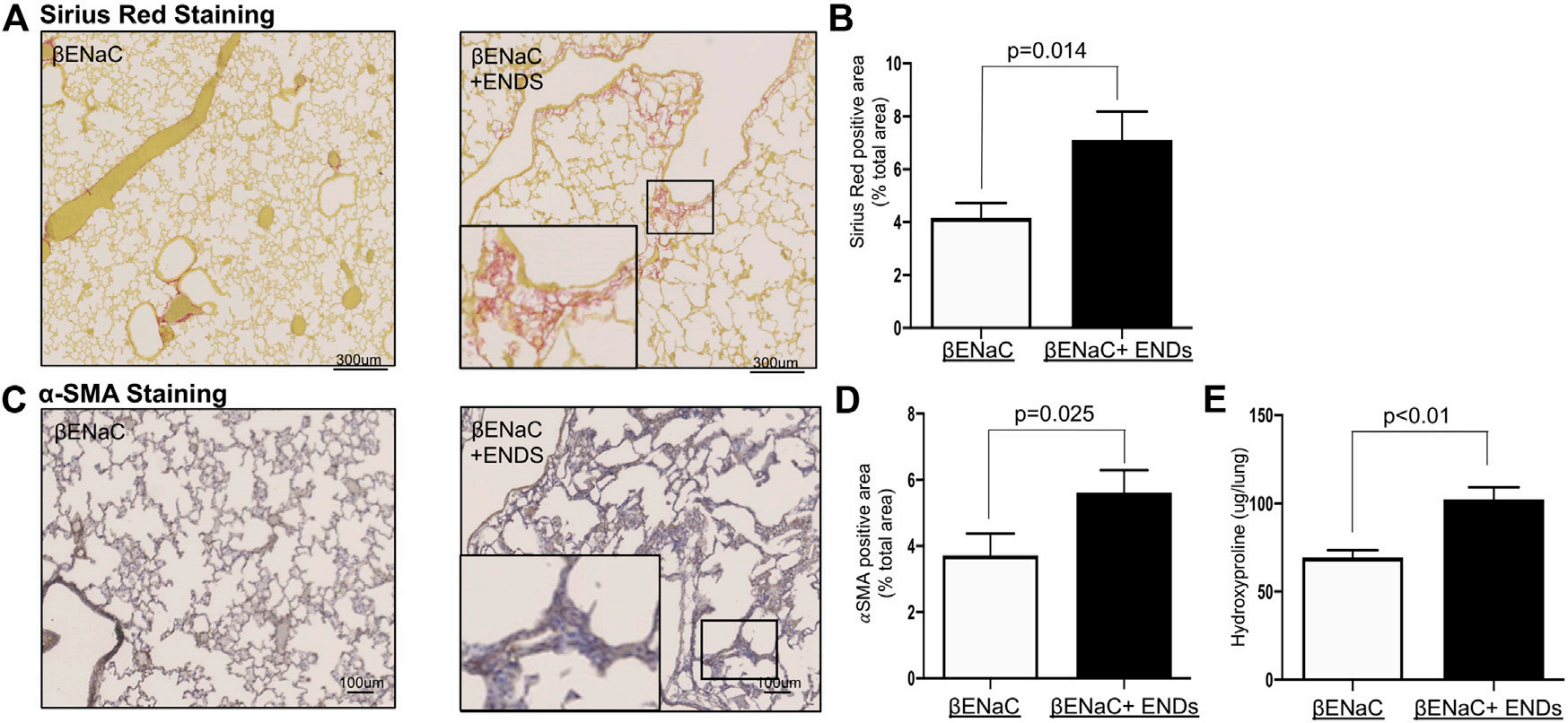
E-cigarette exposure enhances fibrosis in the lungs. **(A)** Representative images of Pico-Sirius Red staining in lung sections from βENaC mice with or without ENDS exposure (*n* = 4 per group). The inset indicates the enlarged image of the boxed area showing positive staining. **(B)** Quantitative analysis of Pico-Sirius Red staining as in **(A)**. The positive staining areas were calculated as percentages of total area, using five randomly selected sections per mouse, a total of four mice in each group. **(C)** Representative images of α-SMA IHC staining in lung sections from βENaC mice with or without ENDS exposure (*n* = 4 per group). The inset indicates the enlarged image of the boxed area showing positive staining. **(D)** Quantitative analysis of α-SMA IHC staining as in **(C)**. The positive staining areas were calculated as percentages of total area, using five randomly selected sections per mouse, a total of four mice in each group. **(E)** Measurement of collagen contents by hydroxyproline assay using lung tissues of βENaC mice with or without ENDS exposure (*n* = 4 per group). Columns and error bars represent means ± SEM.

Substantial studies have shown that M2 macrophages promote lung fibrosis (Song et al., 2000; Bargagli et al., 2011; Hou et al., 2018); and ENDS elevate extracellular matrix accumulation and profibrotic progress (Chapman et al., 2019; Shi et al., 2019; Wang et al., 2019; Wang et al., 2020), we next questioned whether fibrotic phenotypes were enhanced in the lungs after exposure to ENDS. We assessed collagen content using Sirius Red staining and found that ENDS exposure dramatically increased collagen levels in mice (Figures 5A,B). The collagen is predominantly produced by myofibroblasts, a group of key effector cells in fibrosis progression which is considered as a hallmark of fibrosis (Hinz, 2007; Hinz et al., 2007; Kendall and Feghali-Bostwick, 2014). We then stained myofibroblasts in lung tissues from mice with or without ENDS delivery, using a commonly used marker α-Smooth Muscle Actin (α-SMA). The result showed that ENDS treatment increased the α-SMA^+^ myofibroblast population in the lungs (Figures 5C,D). In addition, the hydroxyproline contents were also remarkably increased in mice receiving ENDS treatment (Figure 5E). These results demonstrate that ENDS enhance lung fibrotic phenotypes in COPD mice.

## Discussion

Smoking is the leading cause of COPD, which accounts for as much as 90% of COPD risk. ENDS are considered as a safe replacement for cigarette smoking. However, increasing concerns have been raised in recent years as ENDS use shows a high correlation with COPD occurrence (Bowler et al., 2017; Perez et al., 2019), the underneath mechanisms of which have not been fully understood. Here we report that ENDS rewired cytokine production in lungs of COPD mice caused by ENDS exposure. Increasing evidence indicates that chronic inflammatory and immune responses play key roles in the development and progression of COPD (Rovina et al., 2013). Our study is the first one to elucidate that ENDS shaped an anti-inflammatory environment by increasing levels of several important anti-inflammatory cytokines and augmenting the population of the anti-inflammatory M2 phenotype of macrophages. We also found that ENDS increased fibrosis in COPD mice. It has been reported that the development of fibrosis in COPD results in a reduction in lung elasticity, which further worsens COPD (Rovina et al., 2013).

We demonstrated that ENDS exposure elevated inflammation response and enhanced lung fibrosis. Lung inflammation and fibrosis are both essentially implicated in the development and progression of COPD; and they also are closed correlated (Ward and Hunninghake, 1998; Rao et al., 2020). Observations that lung tissues from patients with lung fibrosis display significant inflammation are widely documented, suggesting that the inflammatory process could result in lung fibrosis (Ward and Hunninghake, 1998; Bringardner et al., 2008). Intensive investigations reveal that fibrosis seems to be the end outcome of unresolved chronic inflammatory reactions induced by a variety of stimuli and tissue injury (Wynn, 2008). Specifically, in COPD, the pathogenesis usually starts with an inflammatory process (Cornwell et al., 2010). Under certain circumstances, inflammation reactions are not resolved. Such a chronic, persistent inflammatory response results in extensive fibrosis, and consequently, causes an exacerbation of COPD conditions (Barnes, 2014; Barnes, 2019). In the current study, we found that ENDS exposure elevated inflammation response by increasing multiple cytokine production and enhanced pro-fibrotic activity by augmenting M2 macrophage numbers, both of which worsen the COPD symptoms in mice.

We observed ENDS exposure remarkedly elevated the population of M2 macrophages. The balance of M1/M2 macrophages is well-recognized to control the fate of an organ in inflammation or fibrosis (Patel et al., 2017; Chen et al., 2020). M1 macrophages are known as a pro-inflammatory or anti-fibrotic phenotype as they trigger inflammatory responses by producing proinflammatory cytokines and contribute to tissue destruction (Byrne et al., 2016). Conversely, M2 macrophages are known as an anti-inflammatory or pro-fibrotic phenotype, which are implicated in the aberrant wound-healing process during fibrosis by producing pro-fibrotic cytokines (Byrne et al., 2016; Shapouri-Moghaddam et al., 2018). M2 macrophage numbers are reported to be elevated during fibrotic disease; and excessive M2 macrophages contribute to a pathological fibroproliferative response and consequently promote lung fibrosis found in the later phase of acute lung injury and acute respiratory distress syndrome (Janssen et al., 2011; Xiang et al., 2016). Our finding showing that ENDS elevated M2 macrophage numbers suggests ENDS could lead to a fibrotic amplification which may exacerbate COPD in patients.

We further intended to uncover the underlying mechanisms of how ENDS promoted the alteration of macrophage phenotype. We asked whether ENDS directly regulated the M2 phenotype switch. ENDS is commonly made up of four basic ingredients: water, nicotine, flavorings, and propylene glycol (PG) and/or vegetable glycerin base (VG). Nicotine is usually thought to be the major toxic component in ENDS. It has been shown that cigarette smoke extract (CSE) and pure nicotine can induce M2 phenotype change (Lu et al., 2017; Alqasrawi et al., 2020), so we hypothesized that ENDS may directly induce macrophage M2 polarization *via* nicotine. However, murine macrophage Raw264.7 did not display an increase in Arginase 1 expression level after the treatment of nicotine-containing ENDS juice, which means the ENDS might not directly induce macrophage M2 polarization. This suggests the importance of vaping smoking in causing the M2 macrophage phenotype. Our results elucidated that anti-inflammatory cytokines were increased after ENDS delivery. Such change in cytokines profile is reported as a stimulus to induce macrophage polarization in murine models of emphysema (Kohler et al., 2019). So, the change in cytokine profile, instead of being a consequence of M2 macrophage polarization, might be the cause of M2 macrophage phenotype change.

Myofibroblasts are major effector cells during lung injury and repair. Our results showed that ENDS exposure increased the number of α-SMA^+^ myofibroblasts. The crosstalk between myofibroblast and macrophages has been well-recognized in fibrotic diseases and cancer (Comito et al., 2014; Van Linthout et al., 2014; Fernando et al., 2016; Hou et al., 2018; Johnson et al., 2018; An et al., 2020). Macrophages, especially M2 macrophages release abundant TGF-β1 which is a major driver of the activation of myofibroblasts (Hou et al., 2018). On the other hand, myofibroblasts are active in the secretion of a number of cytokines and chemokines, which are reported to enhance the recruitment and activity of macrophages (Bagalad et al., 2017; Monteran and Erez, 2019). Therefore, another plausible mechanism is that ENDS altered macrophage phenotype through myofibroblast-secreted mediators indirectly, which needs further investigations.

Besides, a typical feature of widespread peribronchiolar fibrosis with collagen deposition in COPD patients suggests fibrosis is probably the earliest and an important mechanism for COPD progression, which however has been long neglected with very few studies (Mcdonough et al., 2011; Hogg et al., 2017; Koo et al., 2018; Barnes, 2019). In this study, we have also investigated fibrotic phenotypes in mice after ENDS exposure. We observed a significant increase in collagen accumulation and α-SMA positive myofibroblast population, indicating ENDS enhanced fibrotic phenotypes in COPD mice. It is plausible that the increase in M2 macrophages induced by ENDS results in the augment of fibrotic phenotypes.

In addition, a notable phenomenon in our study is that ENDS remarkably enriched the myofibroblast population, suggesting ENDS might induce the activation of myofibroblasts. The myofibroblast is the predominant cell type that is responsible for the deposition of extracellular matrix and is considered as a hallmark of fibrotic diseases (Hinz et al., 2007; Hinz, 2016; Pakshir et al., 2020). And we observed lung fibrosis was enhanced in mice who received ENDS exposure. It would be interesting to understand whether ENDS can stimulate the differentiation of fibroblasts to myofibroblasts. This might uncover another potential risk of ENDS as it might promote lung fibrosis development *via* inducing myofibroblasts differentiation.

In conclusion, our study elucidates that ENDS exposure elevates COPD features and causes inflammatory response alteration with an increase in M2 macrophage number. The changes in inflammatory response resulted from ENDS further lead to an enhancement in lung fibrosis. Our study provides us a deeper understanding of the mechanism how ENDS affects COPD. Lastly, though βENaC-overexpressing mice serve as a good species exposed to ENDS for induction of animal model of COPD, the effect of ENDS on wild type mice still requires further investigations.

## Data Availability

The original contributions presented in the study are included in the article/Supplementary Material, further inquiries can be directed to the corresponding author.
